# Real-world outcomes following PARP inhibitor maintenance in ovarian cancer by *BRCA* status: a retrospective cohort study

**DOI:** 10.1016/j.esmorw.2025.100659

**Published:** 2026-01-06

**Authors:** K. Zucker, B. Pickwell-Smith, A. Samani, A. Sujenthiran, H. Pittell, P. Mpofu, G. Hall

**Affiliations:** 1 Leeds Cancer Centre, Leeds, UK; 2 Leeds Institute of Medical Research, University of Leeds, Leeds, UK; 3 Flatiron Health, New York, USA

**Keywords:** ovarian cancer, PARP inhibitors, treatment-free interval, time to next treatment, real-world data, platinum-based chemotherapy

## Abstract

**Background:**

The introduction of poly (adenosine-diphosphate ribose) polymerase inhibitors (PARPis) has significantly improved progression-free survival for patients with ovarian cancer after response to first-line platinum-based chemotherapy. Yet, there are concerns that PARPi may compromise response to further platinum due to cross-resistance mechanisms. This study investigates the clinical effectiveness of chemotherapy post-progression on PARPi maintenance therapy after initial platinum-based treatment for ovarian cancer, regardless of *BRCA* mutations, using real-world data. Additionally, this study summarises results across randomised trials of PARPi as first-line maintenance.

**Materials and methods:**

This study used a United States-based electronic health record-derived de-identified database. A retrospective descriptive analysis was conducted on patients with ovarian cancer diagnosed from 1 January 2015 onwards. Patient data were collected, including *BRCA* and homologous recombination deficiency (HRD) status. Time to next treatment (TTNT), treatment-free interval (TFI), and the impact of *BRCA* and HRD status were assessed.

**Results:**

Among 3649 patients, 81% had known *BRCA* status, of whom 83% were *BRCA*-negative, 17% were *BRCA*-positive, and 19% had unknown *BRCA* status. The majority (80%) had unknown HRD status. Notably, 17% received first-line PARPi. Patients with *BRCA* mutations displayed longer TTNT and TFI when receiving first-line PARPi initially. However, after receiving subsequent treatments, *BRCA*-mutated and non-mutated patients demonstrated shorter TFI and TTNT intervals, suggesting a possible influence of PARPis on subsequent chemotherapy efficacy.

**Conclusions:**

The study underscores previous concerns that initiating PARPi in the first line may impact treatment duration and intervals for subsequent therapies in patients with ovarian cancer. Future investigations should explore the interplay of PARPi maintenance in the first-line setting, HRD status, and response to subsequent platinum-based therapies.

## Highlights

• Subsequent treatment responses are shorter after first-line PARPi in ovarian cancer.
• Shortened responses occur in both second- to third- and third- to fourth-line treatments.
• Shortened effects of subsequent treatment occur irrespective of *BRCA* status.
• Later effects of first-line PARPi by HRD status are unclear due to small numbers.

## Introduction

Ovarian cancer is the second-highest cause of mortality among gynaecological cancers,^1^ with 14 000 deaths in the United States and 26 221 in Europe in 2017.^2^^,^^3^ Most patients are diagnosed at an advanced stage, with high-grade serous carcinoma (HGSC) being the most prevalent subtype.^4^ Approximately 50% of HGSC cancers are associated with homologous recombination deficiency (HRD).^5^ Less common epithelial subtypes include high-grade endometrioid carcinoma, low-grade serous carcinoma, and clear-cell carcinoma.

Despite optimal debulking surgery and platinum-based chemotherapy, many patients relapse within 18 months of first-line treatment.^6^ Progression-free survival (PFS) captures the interval during and after treatment when cancer remains stable. Poly (adenosine-diphosphate ribose) polymerase (PARP) inhibitors (PARPis) have transformed outcomes, with trials demonstrating significant improvements in PFS through maintenance therapy.^7^

Initially used in relapsed cases, PARPis benefit patients with *BRCA* mutations, and HRD-positive and HRD-negative disease. SOLO2 showed significant PFS improvement with olaparib in *BRCA*-mutated patients after two prior chemotherapy lines.^8^ The NOVA trial demonstrated PFS benefit with niraparib, irrespective of *BRCA* or HRD status after two treatment lines.^9^ Meanwhile, clinical trials have explored the benefits of PARPis following first-line chemotherapy, demonstrating significantly prolonged PFS, particularly in patients with a *BRCA* mutation (germline or somatic) and in those with *BRCA* wild-type HRD-positive tumours. Notably, four trials have assessed the efficacy of first-line PARPi as maintenance monotherapy—SOLO-1, PRIMA, VELIA/GOG-3005 and ATHENA-MONO—while one trial has assessed the efficacy of PARPi in combination with bevacizumab as maintenance therapy in the first-line setting (Table 1).^10^, ^12^, ^14^, ^15^, ^16^

**Table 1:** Summary of randomised trials of PARPis as first-line maintenance

Trial (year/s)	Comparison	Progression-free survival (HRD ± *BRCA* mutations)	Progression-free survival (other analyses)	Overall survival (HRD ± *BRCA* mutations)	Overall survival (other analyses)
SOLO-1 ^10^^,^^11^ (2018, 2022)	Olaparib versus placebo	*BRCA* mutation only At 3 years 60% versus 27% HR 0.3, CI 0.23-0.41	NA	*BRCA* mutation only NR versus 75.2 months HR 0.55, CI 0.40-0.76	NA
PRIMA ^12^^,^^13^ (2019)	Niraparib versus placebo	*BRCA*-positive subgroup 22.1 versus 10.9 months HR 0.40, CI 0.27-0.62 HRD-positive subgroup 19.6 versus 8.2 months HR 0.50, CI 0.31-0.83	HRD-negative subgroup 8.1 versus 5.4 months HR 0.68, CI 0.49-0.94	HRD-positive subgroup HR 0.95, CI 0.70-1.29	Overall population OS HR 1.01, CI 0.84-1.23
VELIA ^14^ (2019)	Veliparib versus placebo	*BRCA*-positive subgroup 34.7 versus 22.0 months HR 0.44, CI 0.28-0.68 HRD-positive subgroup 31.9 versus 20.5 months HR 0.57, CI 0.43-0.76	Intention-to-treat 15.2 versus 17.3 months HR 1.07, CI 0.90-1.29	Immature	Immature
ATHENA-MONO ^15^ (2022)	Rucaparib versus placebo	HRD-positive 28.7 versus 11.3 months HR 0.47, CI 0.31-0.72	HRD-negative 12.1 versus 9.1 months HR 0.65, CI 0.45-0.95	Immature	Immature
PAOLA-1 ^16^^,^^17^ (2019, 2023)	Olaparib and bevacizumab versus bevacizumab and placebo	*BRCA* mutation 37.2 versus 21.7 months HR 0.31, CI 0.20-0.47 *BRCA*-negative HRD-positive 28.1 versus 16.6 months HR 0.43, CI 0.28-0.66	HRD-negative or HRD unknown 16.9 versus 16 months HR 0.92, CI 0.72-1.17	*BRCA* mutation At 5 years 73.2% versus 53.8% alive HR 0.60, CI 0.39-0.93 *BRCA*-negative HRD-positive At 5 years 54.7% versus 44.2% alive HR 0.71, CI 0.45-1.13	HRD-negative At 5 years 25.7% versus 32.3% alive HR 1.19, CI 0.88-1.63

CI, confidence interval; HR, hazard ratio; HRD, homologous recombination deficiency; NR, not reached; OS, overall survival; NA, not applicable/not available; PARPi, poly (adenosine-diphosphate ribose) polymerase inhibitor.

SOLO-1 randomised patients diagnosed with stage III or IV high-grade serous or endometrioid ovarian cancer with germline *BRCA* mutations who responded to first-line platinum-based therapy to olaparib or placebo.^10^ Results indicated a significant improvement in PFS. Overall survival (OS) did not reach statistical significance, though it was clinically meaningful (Table 1).^10^^,^^11^

Conversely, the PRIMA trial evaluated the impact of niraparib as maintenance in patients with stage III and IV ovarian cancer after first-line platinum-based chemotherapy without restriction based on HRD status. It revealed a significant PFS benefit with niraparib compared with placebo, irrespective of HRD status. However, the observed benefit was more pronounced in *BRCA*-mutated or *BRCA*-wild-type HRD-positive tumours.^12^ Meanwhile, OS data remain unpublished in peer-reviewed journals but suggest no OS benefit.^18^^,^^13^ Similarly, the VELIA/GOG-3005 trial randomised patients with stage III or IV ovarian cancer, regardless of *BRCA* or HRD status, to receive chemotherapy alone or chemotherapy plus veliparib followed by veliparib maintenance or placebo. While there were significant improvements in PFS for those with *BRCA* mutations and HRD positivity, the benefit was not statistically significant in the HRD-negative cohort (Table 1).^14^

Meanwhile, the PAOLA-1/ENGOT-ov25 trial included patients with stage III and IV ovarian cancer, irrespective of *BRCA* or HRD status, post-initial platinum-based therapy, randomising to maintenance with olaparib plus bevacizumab or placebo plus bevacizumab. In the HRD-positive population, the combination of olaparib and bevacizumab significantly improved PFS and OS. However, these results were not replicated in HRD-negative tumours.^16^^,^^17^ Finally, the ATHENA-MONO trial compared rucaparib versus placebo in patients with stage III-IV high-grade cancer following response to first-line platinum-based chemotherapy, stratified by HRD status. Rucaparib improved PFS for patients with and without HRD (Table 1).^15^

These studies demonstrated PFS benefits in HRD-positive patients receiving first-line PARPi. However, results for HRD-negative patients are mixed, with varying PFS outcomes, and no OS advantage in HRD-negative, *BRCA*-wild-type HRD-positive, or HRD-positive groups.^12^^,^^17^ Substantial OS benefits were seen in *BRCA*-mutated patients in SOLO1 and PAOLA-1, despite 44.3%-45.7% crossing over in the placebo group.^11^^,^^17^

Given the lack of survival benefits in *BRCA*-negative HRD-positive, or HRD-negative groups, additional evidence is needed to identify who benefits from PARPi treatment and the optimal treatment timing.

Emerging evidence suggests PARPis may drive resistance to subsequent treatment. Cecere et al. reported unexpectedly low response rates after olaparib maintenance therapy.^19^ Baert et al. reported reduced platinum response in the third-line setting in patients after PARPi.^20^

The SOLO2 *post hoc* analysis showed a 7.3-month longer time to second progression in the placebo group.^21^ Cleveland Clinic data showed significantly worse PFS after PARPi in *BRCA*-mutated patients in the second- or third-line setting (8.0 months versus 19.1 months).^22^ Retrospective analysis by Romeo et al. reported that the response rates to subsequent platinum-based chemotherapy were worse among patients with *BRCA* mutations than patients with *BRCA* wild-type.^23^ Collectively, these studies suggest a potential role of PARPis in promoting platinum resistance in the relapsed setting. Suggested mechanisms for this potential cross-resistance have also been postulated.^24^ These concerns highlight a need for more comprehensive real-world studies to understand the impact of PARPi maintenance, particularly in the first-line setting where the evidence remains scarce.

The current study aims to describe the efficacy of chemotherapy following disease progression on PARPi maintenance therapy in the first-line setting, irrespective of *BRCA* mutational status, leveraging real-world data sources.

## Materials and methods

### Study type and dataset

This retrospective observational descriptive real-world data study was delivered utilising the Flatiron Health database, a longitudinal database comprising de-identified patient-level structured and unstructured data curated via technology-enabled abstraction.^25^^,^^26^ During the study period, the de-identified data originated from ∼280 United States cancer clinics (∼800 care sites). Most patients in the database originate from community oncology settings; relative community/academic proportions may vary by cohort. The study included patients diagnosed with primary ovarian, fallopian tube, and primary peritoneal cancers from 1 January 2015 to 1 July 2023.

### Inclusion criteria and cohort design

As this study relies on the Flatiron Health ovarian dataset, patients were required to meet two sets of inclusion criteria: those that determine inclusion in the original source dataset and a subsequent set of study-specific criteria.

#### Flatiron Health source data inclusion criteria

The Flatiron datamart-specific inclusion criteria were as follows:

• Diagnosis with an International Classification of Diseases (ICD) code for ovarian, fallopian tube, or peritoneal cancer (ICD 9: 183x, 158x; ICD 10: C56x, C57.0x, C48x) on or after 1 January 2011.
• Histology: one of serous, mucinous, clear cell, transitional cell, endometrioid, epithelial not otherwise specified (NOS), borderline, and unknown/undocumented. Note: High- versus low-grade serous distinction was unavailable in this dataset.
• At least two documented clinical visits, on different days, occurring on or after 1 January 2011. This was a pragmatic decision to ensure sufficient clinical data.

#### Study-specific inclusion criteria and cohort design

After receiving the data, further inclusion criteria were applied. Patients were limited to those diagnosed from 1 January 2015 to 1 July 2023 with evidence of receiving platinum-based therapy in the first-line (non-maintenance) setting. Patients with borderline, mucinous, transitional cell, or unknown histology were excluded as PARPi is not routinely used in these subtypes. To mitigate issues of immortal time bias, patients were also limited to those receiving at least six cycles of platinum-based chemotherapy in the first-line setting. This issue required addressing due to the use of post-baseline information (use of maintenance PARPi) to classify individuals at baseline.

Sub-cohorts were created based on treatment and molecular status. This first sub-cohort division was based on treatment, and the total study population was divided into two groups:

1) A first-line PARPi group, including patients treated with first-line PARPi maintenance therapy (olaparib, niraparib, or rucaparib).
2) A non-first-line PARPi group, including all patients with no evidence of maintenance therapy or non-maintenance therapy with a PARPi (olaparib, niraparib, or rucaparib) as part of their first-line treatment.

These cohorts were then subdivided based on molecular status, dividing each group further by *BRCA*-positive and *BRCA*-negative status. *BRCA* positivity was defined as the presence of either a germline or somatic *BRCA* mutation. Patients with unknown molecular status were excluded from these sub-cohorts, while those with a *BRCA* variant of unknown significance were treated as *BRCA*-negative. *BRCA* positivity was defined as those with recorded evidence of a pathological mutation of *BRCA1* and/or *BRCA2* in either the germline or somatic testing.

A further tier of subdivision was then applied based on HRD molecular status. This resulted in sub-cohorts based on *BRCA* status, HRD status, and PARPi treatment.

### Baseline characteristics

Summary information relating to baseline characteristics was derived for each patient cohort. This included information on the baseline Eastern Cooperative Oncology Group (ECOG), race/ethnicity, age, socioeconomic status, and International Federation of Gynecology and Obstetrics (FIGO) stage. Where patients had multiple ECOG scores recorded, the score closest in number of days to the date of diagnosis was used. Only ECOG recorded within 30 days before or after diagnosis were included with those patients without an ECOG score recorded within this window marked as ECOG unknown.

### Time-to-event analysis

**Figure 1:**
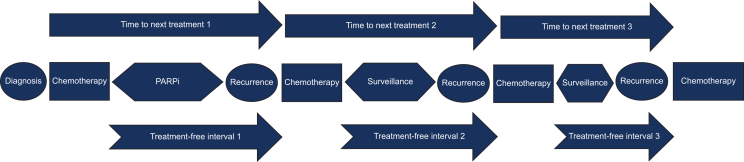
**Time to next treatment and treatment-free intervals definitions and visual explanations.** PARPi, poly (adenosine-diphosphate ribose) polymerase inhibitors.

The study focused on describing several time-to-event metrics across the total population and pre-specified sub-populations. These included OS, time to next treatment (TTNT) for the second, third and fourth treatment, and treatment-free interval (TFI) after the first, second and third treatment. OS was defined as the period from diagnosis until the date of death or censoring. The TFI was defined as the time from the last dose of non-maintenance systemic anticancer treatment (SACT) until the next line of systemic anticancer treatment. TTNT one (TTNT1) was defined as the time from starting the first line of non-maintenance SACT until the start of the second subsequent line of SACT (Figure 1). TTNT two (TTNT2) was defined as the time from completion of the second line of chemotherapy to the initiation of third-line chemotherapy. TTNT three (TTNT3) was defined as the time from completion of the third course of chemotherapy to the initiation of fourth-line chemotherapy. A new line of therapy was defined according to Flatiron Health’s oncologist-defined, rule-based lines of therapy.

Because death dates are provided only as month and year (for privacy), the date of death was estimated as the 15th of the death month unless post-death encounters were recorded—in which case the last-seen date was used. This avoided negative time at risk and affected only a few patients. Each time-to-event analysis was conducted using a Kaplan–Meier estimator in the relevant population. All analyses were undertaken using R version 4.2.1 with packages used detailed in Supplementary Table S1, available at https://doi.org/10.1016/j.esmorw.2025.10065.

As the dataset did not contain information relating to the histological grade of each patient’s tumour which is directly related to the likelihood of receiving PARPi, only descriptive analysis was undertaken and no comparative statistical tests have been applied.

### Ethical approval

The local institutional ethics panel had granted approval (MREC 23-018). Prior institutional review board (IRB) approval with included waiver of informed consent was obtained. The WCG IRB granted approval. (The Flatiron Health Real-World Evidence Parent Protocol, Tracking # FLI118044). ESMO GROW checklist can be found in the Supplementary Material, available at https://doi.org/10.1016/j.esmorw.2025.10065.

## Results

**Figure 2:**
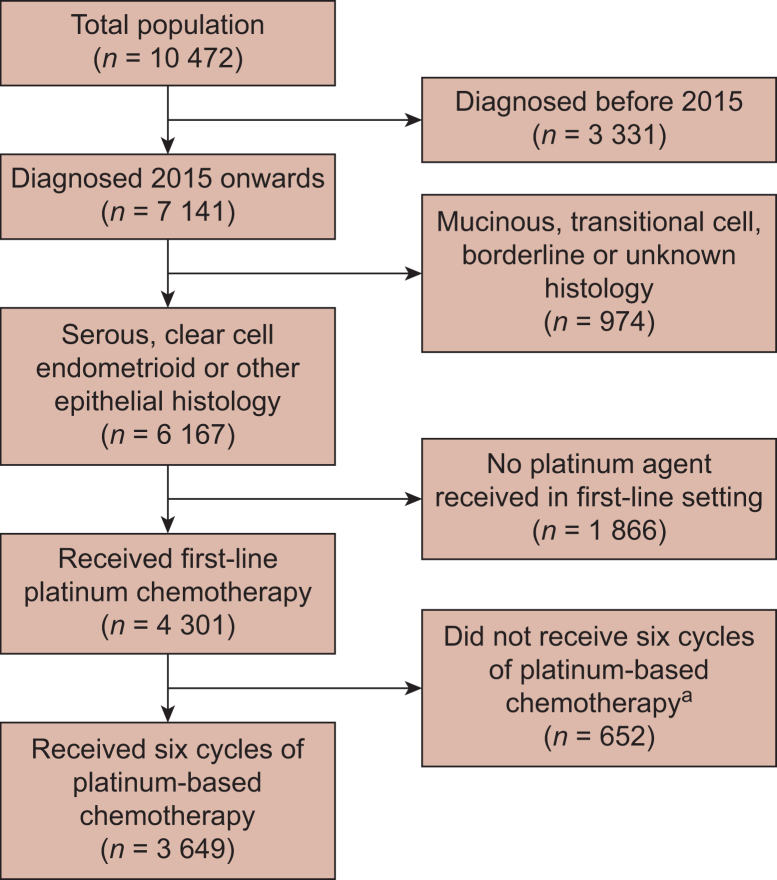
**Flow diagram to show the exclusions applied to the whole cohort.**^a^Numbers may reflect that some patients were still receiving treatment at the data cut-off point.

This study involved 280 United States clinics and identified 3649 patients who met the eligibility criteria (Figure 2). The median age at diagnosis was 66 years (interquartile range 57-73 years). Sixty-four percent of patients were white, 8.6% were Latin, 5.7% were black or African American, 3% were Asian, and 9.1% were other specified ethnicities, with 9.5% unknown. Patient demographics, tumour subtypes, and characteristics are summarised in Table 2. The median duration of follow-up was 30 months.

**Table 2:** Patient characteristics, stage, histology, and HRD status grouped by receipt of PARPi use in first-line setting or not

Characteristic	All patients *N* = 3649	First-line PARPi *N* = 621	*BRCA*-negative first-line PARPi, *N* = 376	*BRCA*-positive first-line PARPi, *N* = 202	*BRCA* unknown first-line PARPi, *N* = 43	Non-first-line PARPi, *N* = 3028	*BRCA*-negative non-first-line PARPi, *N* = 2093	*BRCA*-positive non-first-line PARPi, *N* = 297	*BRCA* unknown non-first-line PARPi, *N* = 638
Age at diagnosis, years (range)	66 (57-73)	65 (57-72)	66 (60-73)	61 (53-69)	64 (57-77)	66 (57-74)	66 (57-73)	61 (54-68)	68 (59-77)
Deprivation, *n* (%)									
1 (most deprived)	464 (13)	71 (11)	42 (11)	19 (9.4)	≤10 (23)	393 (13)	245 (12)	45 (15)	103 (16)
2	570 (16)	102 (16)	64 (17)	30 (15)	≤10 (23)	468 (15)	330 (16)	39 (13)	99 (16)
3	672 (18)	112 (18)	72 (19)	35 (17)	≤10 (23)	560 (18)	379 (18)	54 (18)	127 (20)
4	818 (22)	150 (24)	89 (24)	54 (27)	≤10 (23)	668 (22)	468 (22)	73 (25)	127 (20)
5 (least deprived)	847 (23)	141 (23)	86 (23)	48 (24)	≤10 (23)	706 (23)	500 (24)	72 (24)	134 (21)
Unknown	278 (7.6)	45 (7.2)	23 (6.1)	16 (7.9)	≤10 (23)	233 (7.7)	171 (8.2)	14 (4.7)	48 (7.5)
ECOG, *n* (%)									
0	557 (15)	119 (19)	68 (18)	45 (22)	≤10 (23)	438 (14)	326 (16)	44 (15)	68 (11)
1	495 (14)	89 (14)	55 (15)	28 (14)	≤10 (23)	406 (13)	264 (13)	48 (16)	94 (15)
2	119 (3.3)	22 (3.5)	15 (4.0)	≤10 (4)	≤10 (23)	97 (3.2)	67 (3.2)	≤10 (3)	22 (3.4)
3	34 (0.9)	≤10 (2)	≤10 (3)	≤10 (4)	≤10 (23)	28 (0.9)	18 (0.9)	≤10 (3)	≤10 (2)
4	≤10 (<0.1)	≤10 (2)	0 (0)	≤10 (4)	0 (0)	0 (0)	0 (0)	0 (0)	0 (0)
Unknown	≥2434 (67)	≥381 (62)	≥228 (61)	123 (61)	27 (63)	2059 (68)	1418 (68)	195 (66)	≥444 (70)
Race/ethnicity, *n* (%)									
Latin	312 (8.6)	50 (8.1)	23 (6.1)	19 (9.4)	≤10 (23)	262 (8.7)	163 (7.8)	25 (8.4)	74 (12)
Asian	110 (3.0)	26 (4.2)	17 (4.5)	≤10 (4)	≤10 (23)	84 (2.8)	57 (2.7)	12 (4.0)	15 (2.4)
Black or African American	208 (5.7)	48 (7.7)	29 (7.7)	15 (7.4)	≤10 (23)	160 (5.3)	93 (4.4)	17 (5.7)	50 (7.8)
Other race	333 (9.1)	51 (8.2)	36 (9.6)	13 (6.4)	≤10 (23)	282 (9.3)	198 (9.5)	24 (8.1)	60 (9.4)
White	2339 (64)	384 (62)	227 (60)	≥128 (63)	26 (60)	1955 (65)	1385 (66)	190 (64)	380 (60)
Unknown	347 (9.5)	62 (10.0)	44 (12)	17 (8.4)	≤10 (23)	285 (9.4)	197 (9.4)	29 (9.8)	59 (9.2)
Stage at diagnosis, *n* (%)									
0	403 (11)	62 (10.0)	38 (10)	16 (7.9)	≤10 (23)	341 (11)	228 (11)	22 (7.4)	91 (14)
1	477 (13)	17 (2.7)	≤10 (2.7)	≤10 (4)	0 (0)	460 (15)	297 (14)	36 (12)	127 (20)
2	323 (8.9)	31 (5.0)	16 (4.3)	14 (6.9)	≤10 (23)	292 (9.6)	199 (9.5)	29 (9.8)	64 (10)
3	1560 (43)	319 (51)	197 (52)	≥105 (52)	14 (33)	1241 (41)	895 (43)	141 (47)	205 (32)
4	886 (24)	192 (31)	≥115 (31)	57 (28)	20 (47)	694 (23)	474 (23)	69 (23)	151 (24)
Histology, *n* (%)									
Clear cell	235 (6.4)	≤10 (2)	≤10 (2.7)	≤10 (4)	≤10 (23)	227 (7.5)	165 (7.9)	≤10 (3)	60 (9.4)
Endometrioid	288 (7.9)	15 (2.4)	13 (3.5)	≤10 (4)	0 (0)	273 (9.0)	191 (9.1)	≤10 (3)	72 (11)
Epithelial NOS	522 (14)	84 (14)	52 (14)	27 (13)	≤10 (23)	438 (14)	294 (14)	28 (9.4)	116 (18)
Serous	2604 (71)	≥512 (82)	≥301 (81)	≥151 (76)	≥27 (63)	2090 (69)	1443 (69)	257 (87)	390 (61)
*BRCA* status, *n* (%)									
Negative	2469 (68)	376 (61)	376 (100)	0 (0)	0 (0)	2093 (69)	2093 (100)	0 (0)	0 (0)
Positive	499 (14)	202 (33)	0 (0)	202 (100)	0 (0)	297 (10)	0 (0)	297 (100)	0 (0)
Unknown	681 (19)	43 (7)	0 (0)	0 (0)	43 (100)	638 (21)	0 (0)	0 (0)	638 (100)
HRD, *n* (%)									
Negative	363 (9.9)	63 (10)	57 (15)	≤10 (4)	≤10 (23)	300 (9.9)	291 (14)	≤10 (3)	≤10 (2)
Positive	349 (9.6)	156 (25)	101 (27)	52 (26)	≤10 (23)	193 (6.4)	135 (6.5)	53 (18)	≤10 (2)
Unknown	2937 (80)	402 (65)	218 (58)	≥140 (72)	29 (67)	2535 (84)	1667 (80)	≥234 (78)	625 (98)

Where patient numbers where ≤10 data obfuscation has been applied as a privacy preserving measure.

ECOG, Eastern Cooperative Oncology Group; HRD, homologous recombination deficiency; NOS, not otherwise specified; PARPi, poly (adenosine-diphosphate ribose) polymerase inhibitor.

Approximately 71% of patients had serous histology, 14% had epithelial NOS, 7.9% endometrioid, and 6.4% had clear-cell histology. The majority of patients (68%) were *BRCA*-negative, a significant proportion (19%) had an unknown *BRCA* status, and the remainder (14%) were *BRCA*-positive. Most patients (80%) had unknown HRD status, 9.9% of patients were negative for HRD, and 9.6% had HRD-positive tumours.

A total of 621 patients (17%) received first-line maintenance PARPi. Of these, 376 (61%) were *BRCA*-negative, 202 (33%) were *BRCA*-positive, and 43 (7%) were *BRCA* unknown. Meanwhile, 3028 patients (83%) did not receive first-line PARPi. Of these, 2093 (69%) were *BRCA*-negative, 297 (10%) were *BRCA*-positive, and 638 (21%) were *BRCA* unknown. Bevacizumab was received within the first line of treatment by 882 patients of whom 219 were in the cohort receiving PARPi and the remaining 663 were in the non-PARPi-receiving cohort.

### Overall survival

The reverse Kaplan–Meier-derived median study follow-up time from diagnosis until data cut-off was 55.1 months. The median OS was 60 months [95% confidence interval (CI) 57-64 months]. Median OS was higher in patients receiving a first-line PARPi [68 months (95% CI 62-82 months)] compared with patients not receiving a first-line PARPi [58 months (95% CI 56-62 months)]. This higher estimated survival was seen for patients with *BRCA*-positive {>100 [95% CI 68-not reached (NR)] versus 90 [95% CI 85-NR]} and -negative disease [66 (95% CI 50-NR) versus 57 (95% CI 54-61)]; however, variation in precision of estimates due to varying patient numbers was also apparent. However, patients with an unknown *BRCA* status treated with first-line PARPi had a slightly shorter median OS [40 months (95% CI 33 months-NR)] compared with those who did not receive a first-line PARPi [45 months (95% CI 39-54 months)] (Table 3).

**Table 3:** Kaplan–Meier-derived estimates at 1-5 years—results shown for overall survival, time to second, third, and fourth treatment broken down by PARPi and *BRCA* status

Characteristic	Overall survival, years					Time to second treatment, years					Time to third treatment, years					Time to fourth treatment, years				
	1	2	3	4	5	1	2	3	4	5	1	2	3	4	5	1	2	3	4	5
All patients																				
All patients *n* = 3649	95% (94-96)	82% (81-84)	70% (68-71)	59% (57-61)	50% (47-52)	71% (70-73)	42% (40-44)	33% (32-35)	28% (26-30)	24% (23-26)	39% (36-41)	18% (16-20)	11% (9.1-13)	8.4% (6.9-10)	7.1% (5.6-9.0)	26% (24-29)	8.3% (6.6-10)	4.6% (3.2-6.4)	2.8% (1.7-4.8)	1.4% (0.5-4.3)
Receiving first-line PARPi *n* = 621	98% (97-99)	87% (85-90)	74% (69-78)	65% (60-71)	57% (51-65)	86% (83-89)	52% (48-57)	40% (35-45)	32% (27-38)	25% (19-32)	24% (19-32)	10.0% (5.9-17)	7.1% (3.5-14)			14% (8.6-23)	5.2% (2.0-13)	2.6% (0.5-14)	2.6% (0.5-14)	
Not receiving first-line PARPi *n* = 3.028	94% (94-95)	81% (80-83)	69% (67-71)	58% (56-60)	49% (46-51)	68% (66-70)	40% (38-42)	32% (30-34)	27% (25-29)	24% (22-26)	41% (38-43)	19% (17-21)	11% (9.5-13)	8.9% (7.3-11)	7.5% (5.9-9.5)	28% (25-31)	8.7% (6.9-11)	4.8% (3.4-6.8)	2.9% (1.6-5.0)	1.4% (0.5-4.4)
*BRCA*-positive																				
Receiving first-line PARPi *n* = 202	100% (100-100)	96% (93-99)	87% (82-93)	77% (68-86)	72% (62-83)	95% (91-98)	76% (70-83)	63% (55-72)	49% (40-60)	38% (27-53)	26% (15-44)	14% (5.7-34)	14% (5.7-34)			22% (9.9-50)				
Not receiving first-line PARPi *n* = 297	99% (98-100)	96% (94-98)	90% (86-94)	79% (74-85)	70% (64-77)	79% (74-84)	45% (39-52)	35% (30-42)	30% (25-37)	27% (22-34)	66% (59-73)	40% (33-49)	28% (22-37)	25% (18-33)	21% (14-33)	37% (27-49)	12% (6.5-22)	8.8% (4.1-19)	6.6% (2.6-17)	
*BRCA*-negative																				
Receiving first-line PARPi *n* = 376	97% (96-99)	85% (81-89)	68% (62-74)	62% (55-69)	52% (44-63)	82% (78-86)	41% (36-47)	30% (25-36)	25% (19-32)	19% (12-30)	24% (18-33)	9.5% (5.1-18)	4.7% (1.5-15)			11% (5.4-21)	5.4% (1.9-15)	2.7% (0.5-15)	2.7% (0.5-15)	
Not receiving first-line PARPi *n* = 2093	95% (94-96)	83% (81-84)	69% (67-72)	57% (55-60)	48% (45-51)	67% (65-69)	38% (36-41)	30% (28-32)	24% (22-26)	21% (19-23)	39% (36-42)	17% (15-20)	8.9% (7.2-11)	6.5% (4.9-8.7)	5.0% (3.4-7.3)	28% (25-32)	8.7% (6.6-11)	4.9% (3.3-7.3)	2.7% (1.3-5.4)	1.8% (0.6-5.2)
*BRCA* unknown																				
Receiving first-line PARPi *n* = 43	95% (89-100)	71% (58-88)	56% (39-79)	42% (25-71)	31% (14-68)	81% (69-94)	38% (24-59)	14% (5.2-38)	14% (5.2-38)		21% (6.2-68)					30% (6.3-100)				
Not receiving first-line PARPi *n* = 638	90% (88-93)	70% (66-74)	56% (52-61)	49% (44-54)	40% (36-46)	67% (63-71)	44% (40-48)	39% (35-43)	35% (31-39)	31% (27-36)	31% (26-38)	12% (8.3-18)	9.0% (5.7-14)	7.3% (4.3-13)	7.3% (4.3-13)	19% (13-28)	6.0% (2.7-13)			

HRD, homologous recombination deficiency; PARPi, poly (adenosine-diphosphate ribose) polymerase inhibitor.

Expressed as an estimated percentage without the event of interest and the associated 95% confidence interval.

### Treatment cross-over

**Figure 3:**
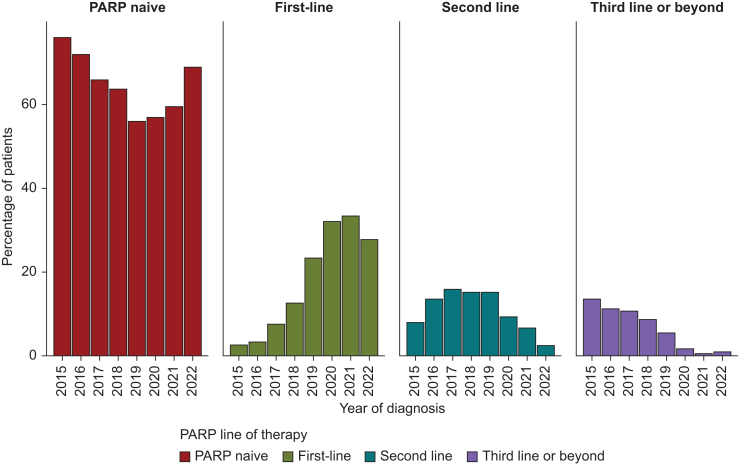
**Proportion of patients receiving PARPi by year of diagnosis and line of therapy—data only included where complete data for the year are available.** PARPi, poly (adenosine-diphosphate ribose) polymerase inhibitors.

Within the non-first-line PARPi cohort, 633 (21%) patients received later-line PARPi: 391 in the second-line setting, 134 in the third-line setting, 60 in the fourth-line setting, and 48 in the beyond-fourth-line setting (Figure 3).

#### Time to next treatment one (TTNT1)

The median TTNT1 for all patients receiving first-line PARPi was 26 months (95% CI 23-29 months), with a median TFI one (TFI1) of 20 months (95% CI 18-24 months). In contrast, patients not receiving first-line PARPi had a median TTNT1 of 18 months (95% CI 17-19 months) and a median TFI1 of 13 months (95% CI 12-14 months). Among patients with a *BRCA* mutation who received first-line PARPi, the median TTNT1 was 48 months (95% CI 42 months-NR), and the median TFI1 was 44 months (95% CI 36 months-NR), compared with 21 months (95% CI 19-26 months) and 16 months (95% CI 14-20 months) for those who did not receive first-line PARPi. Similarly, *BRCA*-negative patients receiving first-line PARPi had a median TTNT1 and TFI1 of 20 months (95% CI 19-23 months) and 14 months (95% CI 13-18 months), respectively, compared with 17 months (95% CI 17-18 months) and 12 months (95% CI 11-13 months) for *BRCA*-negative patients not receiving first-line PARPi. Patients with *BRCA* unknown status also demonstrated slight difference when treated with first-line PARPi, with a median TTNT1 of 19 months (95% CI 17-28 months) compared with 18 months among those not exposed to a first-line PARPi (95% CI 16-21 months) and a median TFI1 of 14 months (95% CI 8.6-23 months) compared with 13 months among those unexposed to first-line PARPi (95% CI 11-17 months).

#### Time to next treatment two (TTNT2)

A total of 1878 patients completed second-line therapy. The median TTNT2 for all patients receiving first-line PARPi was 7.8 months (95% CI 7.1-8.8 months), with a median TFI2 of 2.7 months (95% CI 1.8-3.6 months). In contrast, patients not receiving first-line PARPi had a median TTNT2 of 9.7 months (95% CI 9.2-10 months) and a median TFI2 of 3.9 months (95% 3.4-4.4 months). Among patients with a *BRCA* mutation who received first-line PARPi, the median TTNT2 was 7.6 months (95% CI 6.9-10 months), and the median TFI2 was 2.8 months (95% CI 1.8-5.5 months), compared with 17 months (95% 15-22 months) and 9.7 months (95% CI 7.3-13 months) for those who did not receive first-line PARPi. Similarly, *BRCA*-negative patients receiving first-line PARPi had a median TTNT2 of 7.8 months (95% CI 6.8-8.7 months) and TFI2 of 2.1 months (95% CI 1.4-3.6 months), respectively, compared with 9.4 months (95% CI 8.8-10 months) and 3.7 months (95% CI 3-4.3 months) for *BRCA*-negative patients not receiving first-line PARPi. Meanwhile, patients with *BRCA* unknown seemed to have more extended periods between treatments when receiving first-line PARPi, with a median TTNT2 of 11 months (95% CI 5.6 months-not reached) compared with 7.2 months (95% CI 6-8.6 months) and median TFI2 of 3.8 months (95% CI 2.7 months-not reached) compared with 2.5 months (95% CI 1.7-3.8 months).

#### Time to next treatment three (TTNT3)

A total of 1132 patients completed third-line treatment. The median TTNT3 for all patients receiving first-line PARPi was 4.9 months (95% CI 3.8-5.9 months), with a median TFI3 of 1.1 months (95% CI 0.9-1.5 months). In contrast, patients not receiving first-line PARPi had a median TTNT3 of 6.2 months (95% CI 5.8-6.9 months) and the median TFI3 was 1.7 months (95% CI 1.6-2.1 months). Among patients with a *BRCA*-positive mutation who received first-line PARPi, the median TTNT3 was 5.5 months (95% CI 3.9-14 months), and the median TFT3 was 1.1 months (95% CI 0.8-10 months), compared with 8.7 months (95% CI 6.7-12 months) and 2.3 months (95% CI 1.6-3.5 months) for those who did not receive first-line PARPi. Similarly, *BRCA*-negative patients receiving first-line PARPi had a median TTNT3 and TFI3 of 4.5 months (95% CI 3.2-5.9 months) and 1.1 months (95% CI 0.9-1.6 months), respectively, compared with 6.2 months (95% CI 5.9-6.9 months) and 1.6 months (95% CI 1.5-1.2 months) for *BRCA*-negative patients not receiving first-line PARPi. Meanwhile, there was a more prolonged clinical benefit seen for patients with *BRCA* unknown treated with first-line PARPi, with a median TTNT3 of 10 months (95% CI 3.2 months-NR) compared with 5.5 months (95% CI 4.6-6.5 months) and a median TFI3 of 2.2 months (95% CI 1.2 months-NR) compared with 1.6 months (95% CI 1.5-2.2 months).

### Results by BRCA and HRD status

Further differences in the length of survival were seen when the population was broken down by HRD and *BRCA* status, as shown in Table 4.

**Table 4:** Overall survival and median time to next treatment by *BRCA* and HRD status

Cohort	Overall survival			First to second treatment			Second to third treatment			Third to fourth treatment		
	Number	Number (events)	Median	Number	Number (events)	Median time to next treatment	Number	Number (events)	Median time to next treatment	Number	Number (events)	Median time to next treatment
All patients	3649	1312	60 (57-64)	3647	2190	19 (18-20)	1878	1434	9.4 (9.0-9.9)	1132	922	6.0 (5.7-6.5)
*BRCA*-positive												
Receiving first-line PARPi	52	8	62 (46-not reached)	52	19	31 (23-not reached)	18	13	7.1 (4.2-not reached)	11	8	4.9 (1.9-not reached)
Not receiving first-line PARPi	53	12	89 (89-not reached)	53	35	19 (15, 26)	35	25	20 (15, 30)	22	16	7.4 (5.6, 12)
*BRCA*-negative but HRD-positive												
Receiving first-line PARPi	88	7	>77 (not reached-not reached)	88	37	26 (22-not reached)	35	22	7.4 (6.2-23)	20	13	5.2 (3.1-10)
Not receiving first-line PARPi	129	46	60 (55-74)	129	92	16 (14-20)	87	68	10 (8.6-13)	63	54	6.7 (5.3-9.5)
*BRCA*-negative and HRD-negative												
Receiving first-line PARPi	51	9	49 (49-not reached)	51	32	15 (13-22)	31	19	8.3 (4.9-not reached)	17	11	3.8 (2.9-not reached)
Not receiving first-line PARPi	279	67	61 (50-78)	279	150	19 (16-23)	134	88	10 (8.6-13)	78	61	5.9 (5.1-8.0)

HRD, homologous recombination deficiency; PARPi, poly (adenosine-diphosphate ribose) polymerase inhibitor.

## Discussion

This descriptive study analysed real-world outcomes in women with ovarian cancer who completed six or more cycles of platinum-based chemotherapy with and without first-line PARPi. While clinical trials have shown PARPi efficacy (olaparib, niraparib, and rucaparib) in improving PFS,^10^^,^^18^ these trials involve highly selected patients under strict protocols. Applying these findings to broader populations and determining optimal post-PARPi treatments remains challenging.

Standard care introduces PARPi as maintenance for *BRCA*-mutant high-grade serous, endometrioid or clear-cell ovarian cancers, followed by chemotherapy upon progression. However, concerns exist about shorter intervals between treatments, platinum resistance, and reduced efficacy, possibly due to overlapping resistance mechanisms between PARPis and platinum-based chemotherapy.^19^^,^^21^^,^^27^^,^^28^

Cross-resistance may arise from restoration of homologous recombination,^29^^,^^30^ secondary mutations restoring *BRCA1/2* function,^29^^,^^30^ and enhanced DNA replication fork protection enabling tumour survival from either treatment.^29^ These mechanisms complicate relapse management and emphasise the importance of personalised, genetically informed treatment.

OS is a limited endpoint due to cross-over, post-progression therapy, and attrition. While some trials show OS improvement with PARPis, statistical significance is lacking, especially in some subgroups.^11^, ^16^ The PRIMA and PRIME trials showed no OS benefit^13^^,^^30^ highlighting the need for alternative endpoints and more nuanced evaluation based on molecular status.

Our study observed differences in OS, median TFI, and TTNT in PARPi-treated patients, predominantly in those with *BRCA* mutations. TTNT2 and TTNT3 were shorter in *BRCA*-mutated and wild-type patients receiving first-line PARPi, suggesting potential detriment in subsequent chemotherapy efficacy. Further research into the net benefit of PARPi and its impact on subsequent treatment outcomes is needed.

Results stratified by HRD status were heterogeneous due to the recent adoption of HRD testing and low patient numbers generating uncertain estimates.

Study limitations include missingness in HRD status, small PARPi-treated cohorts, and wide CIs. For example, only 69 out of 202 *BRCA*-positive patients receiving first-line PARPi had events during the first to second treatment interval, versus 190 out of 297 in the non-PARPi group. *BRCA* mutation types (*BRCA1*/*BRCA2*, somatic/germline) were not distinguished, potentially impacting outcomes. Immortal time bias may also skew results, as PARPi patients entered the cohort only after multiple chemo cycles, excluding early poor outcomes and inflating apparent benefit. We mitigated some of this by restricting the comparators to those receiving six platinum-based cycles through reduced cohort size and increased uncertainty. This study remains descriptive due to heterogeneous cohorts and limited data granularity. Lack of morphological details (e.g. high-grade versus low-grade serous) may explain difference in *BRCA* mutation prevalence between the PARPi treated group (13.8%) and PARPi untreated group (2.5%).

Real-world progression detection varies from trial protocols, potentially impacting treatment-free intervals and TTNT estimates. Future research should employ target trial type analysis, adjusting for confounders and expanding sample sizes, particularly for non-*BRCA* patients, to better understand PARPi impact.

Additional resistance factors include timing of progression relative to PARPi therapy and residual disease after surgery. Studies show these influence outcomes reinforcing the need for multi-variable analyses to identify predictors of post-PARPi resistance.^31^, ^32^, ^33^

This study responds to concerns that PARPi may drive platinum resistance, a causal question challenging to answer using observational data. Treatment decisions often reflect patient factors such as fitness, age, and comorbidity, introducing selection bias. Thus, our data cannot definitively explain reduced TTNT and TFI.

Although randomised controlled trials can mitigate bias, cross-over of treatment complicates analysis. This study, the largest real-world evaluation of PARPi-treated ovarian cancer patients to date, found shorter TTNT2, TTNT3, TFI2, and TFI3 in those receiving first-line PARPi. While causality is not proven, the trend is clear and warrants further investigation. We advocate for detailed reporting of post-first-line outcomes in clinical trials to better understand this pattern. Further studies should incorporate broader data including detailed – genetic, biological, and imaging to refine treatment strategies. Multicentre, multinational cohorts will be essential to ensure adequate sample size and representativeness of cohorts.

### Conclusion

First-line PARPi patients had shorter TTNT and TFI intervals with differences by *BRCA* status. The findings suggest PARPi may impact subsequent treatment response and highlight the need for further research.

## References

[1] Sung H., Ferlay J., Siegel R.L. (2021). Global cancer statistics 2020: GLOBOCAN estimates of incidence and mortality worldwide for 36 cancers in 185 countries. CA Cancer J Clin.

[2] Dalmartello M., La Vecchia C., Bertuccio P. (2022). European cancer mortality predictions for the year 2022 with focus on ovarian cancer. Ann Oncol.

[3] Siegel R.L., Miller K.D., Jemal A. (2017). Cancer statistics, 2017. CA Cancer J Clin.

[4] Peres L.C., Cushing-Haugen K.L., Köbel M. (2019). Invasive epithelial ovarian cancer survival by histotype and disease stage. J Natl Cancer Inst.

[5] Konstantinopoulos P.A., Ceccaldi R., Shapiro G.I., D’Andrea A.D. (2015). Homologous recombination deficiency: exploiting the fundamental vulnerability of ovarian cancer. Cancer Discov.

[6] McGuire W.P., Hoskins W.J., Brady M.F. (1996). Cyclophosphamide and cisplatin compared with paclitaxel and cisplatin in patients with stage III and stage IV ovarian cancer. N Engl J Med.

[7] Ledermann J.A., Matias-Guiu X., Amant F. (2024). ESGO–ESMO–ESP consensus conference recommendations on ovarian cancer: pathology and molecular biology and early, advanced and recurrent disease. Ann Oncol.

[8] Pujade-Lauraine E., Ledermann J.A., Selle F. (2017). Olaparib tablets as maintenance therapy in patients with platinum-sensitive, relapsed ovarian cancer and a BRCA1/2 mutation (SOLO2/ENGOT-Ov21): a double-blind, randomised, placebo-controlled, phase 3 trial. Lancet Oncol.

[9] Mirza M.R., Monk B.J., Herrstedt J. (2016). Niraparib maintenance therapy in platinum-sensitive, recurrent ovarian cancer. N Engl J Med.

[10] Moore K., Colombo N., Scambia G. (2018). Maintenance olaparib in patients with newly diagnosed advanced ovarian cancer. N Engl J Med.

[11] DiSilvestro P., Banerjee S., Colombo N. (2022). Overall survival with maintenance olaparib at a 7-year follow-up in patients with newly diagnosed advanced ovarian cancer and a BRCA mutation: the SOLO1/GOG 3004 trial. J Clin Oncol.

[12] González-Martín A., Pothuri B., Vergote I. (2019). Niraparib in patients with newly diagnosed advanced ovarian cancer. N Engl J Med.

[13] González-Martín A., Pothuri B., Barretina-Ginesta M.P. (September 13-17, 2024).

[14] Coleman R.L., Fleming G.F., Brady M.F. (2019). Veliparib with first-line chemotherapy and as maintenance therapy in ovarian cancer. N Engl J Med.

[15] Monk B.J., Parkinson C., Lim M.C. (2022). A randomized, phase III trial to evaluate rucaparib monotherapy as maintenance treatment in patients with newly diagnosed ovarian cancer (ATHENA-MONO/GOG-3020/ENGOT-ov45). J Clin Oncol.

[16] Ray-Coquard I., Pautier P., Pignata S. (2019). Olaparib plus bevacizumab as first-line maintenance in ovarian cancer. N Engl J Med.

[17] Ray-Coquard I., Leary A., Pignata S. (2023). Olaparib plus bevacizumab first-line maintenance in ovarian cancer: final overall survival results from the PAOLA-1/ENGOT-ov25 trial. Ann Oncol.

[18] González-Martín A., Pothuri B., Vergote I. (2023). Progression-free survival and safety at 3.5 years of follow-up: results from the randomised phase 3 PRIMA/ENGOT-OV26/GOG-3012 trial of niraparib maintenance treatment in patients with newly diagnosed ovarian cancer. Eur J Cancer.

[19] Cecere S.C., Giannone G., Salutari V. (2020). Olaparib as maintenance therapy in patients with BRCA 1–2 mutated recurrent platinum sensitive ovarian cancer: real world data and post progression outcome. Gynecol Oncol.

[20] Baert T., Ataseven B., Bommert M. (2020). 828P Expected versus observed response to platinum-based chemotherapy after poly (ADP-ribose) polymerase inhibitor treatment for relapsed ovarian cancer. Ann Oncol.

[21] Frenel J.S., Kim J.W., Aryal N. (2022). Efficacy of subsequent chemotherapy for patients with BRCA1/2-mutated recurrent epithelial ovarian cancer progressing on olaparib versus placebo maintenance: post-hoc analyses of the SOLO2/ENGOT Ov-21 trial. Ann Oncol.

[22] Rose P.G., Yao M., Chambers L.M. (2021). PARP inhibitors decrease response to subsequent platinum-based chemotherapy in patients with BRCA mutated ovarian cancer. Anti-cancer Drugs.

[23] Romeo M., Gil-Martín M., Gaba L. (2022). Multicenter real-world data of subsequent chemotherapy after progression to PARP inhibitors in a maintenance relapse setting. Cancers.

[24] Johnson N., Johnson S.F., Yao W. (2013). Stabilization of mutant BRCA1 protein confers PARP inhibitor and platinum resistance. Proc Natl Acad Sci U S A.

[25] Ma X., Long L., Moon S., Adamson B.J.S., Baxi S.S. (2023). Comparison of population characteristics in real-world clinical oncology databases in the US: Flatiron Health, SEER, and NPCR. medRxiv.

[26] Birnbaum B., Nussbaum N., Seidl-Rathkopf K. (2020).

[27] Lin K.K., Harrell M.I., Oza A.M. (2019). BRCA reversion mutations in circulating tumor DNA predict primary and acquired resistance to the PARP inhibitor rucaparib in high-grade ovarian carcinoma. Cancer Discov.

[28] Patch A.-M., Christie E.L., Etemadmoghadam D. (2015). Whole–genome characterization of chemoresistant ovarian cancer. Nature.

[29] Apelian S., Martincuks A., Whittum M. (2025). PARP inhibitors in ovarian cancer: resistance mechanisms, clinical evidence, and evolving strategies. Biomedicines.

[30] Jiang X., Li X., Li W., Bai H., Zhang Z. (2019). PARP inhibitors in ovarian cancer: sensitivity prediction and resistance mechanisms. J Cell Mol Med.

[31] Li N., Zhu J., Yin R. (2023). Treatment With niraparib maintenance therapy in patients with newly diagnosed advanced ovarian cancer: a phase 3 randomized clinical trial. JAMA Oncol.

[32] Harter P., Marth C., Mouret-Reynier M.A. (2025). Efficacy of subsequent therapies in patients with advanced ovarian cancer who relapse after first-line olaparib maintenance: results of the PAOLA-1/ENGOT-ov25 trial. Ann Oncol.

[33] Cecere S.C., Musacchio L., Bartoletti M. (2021). Cytoreductive surgery followed by chemotherapy and olaparib maintenance in BRCA 1/2 mutated recurrent ovarian cancer: a retrospective MITO group study. Int J Gynecol Cancer.

