# Supplementary material for: Real-world outcomes following PARP inhibitor maintenance in ovarian cancer by BRCA status: a retrospective cohort study
Source: ESMO Real World Data Digit Oncol. 2026 Jan 6;11:100659. doi: 10.1016/j.esmorw.2025.100659 (PMC13040904; doi:10.1016/j.esmorw.2025.100659)
Supplement: Supplementary Table S1 [file mmc1.docx]

# **Appendix/Supplementary**

| **Package** | **Version** |
| --- | --- |
| data.table | 1.14.10 |
| dplyr | 1.1.4 |
| flextable | 0.9.4 |
| ggplot2 | 3.4.4 |
| ggpmisc | 0.5.5 |
| ggsci | 3.0.1 |
| gtsummary | 1.7.2 |
| lubridate | 1.9.3 |
| pacman | 0.5.1 |
| pals | 1.8 |
| plyr | 1.8.9 |
| purrr | 1.0.2 |
| stringr | 1.5.1 |
| survival | 3.4-0 |
| survminer | 0.4.9 |
| tidyr | 1.3.0 |
| tidyselect | 1.2.0 |
| zoo | 1.8-12 |

Supplementary Table 1: List of r packages used in analysis
